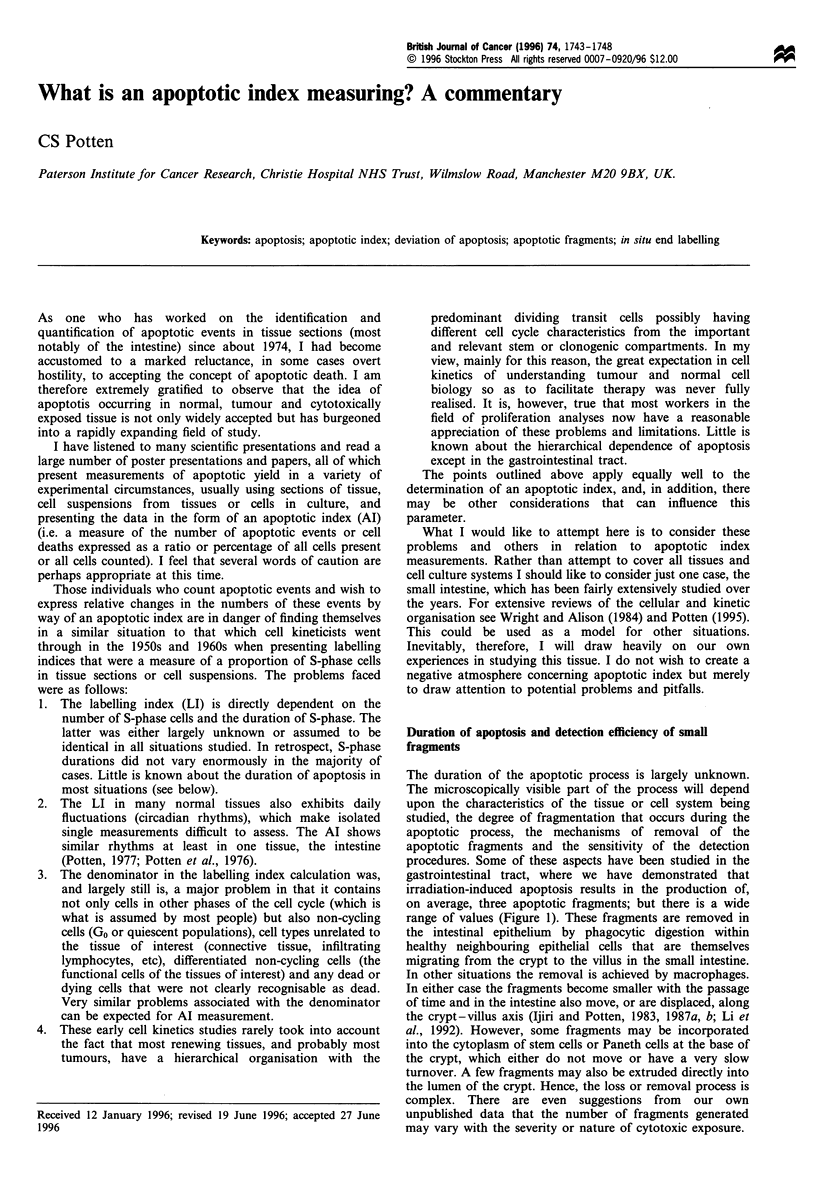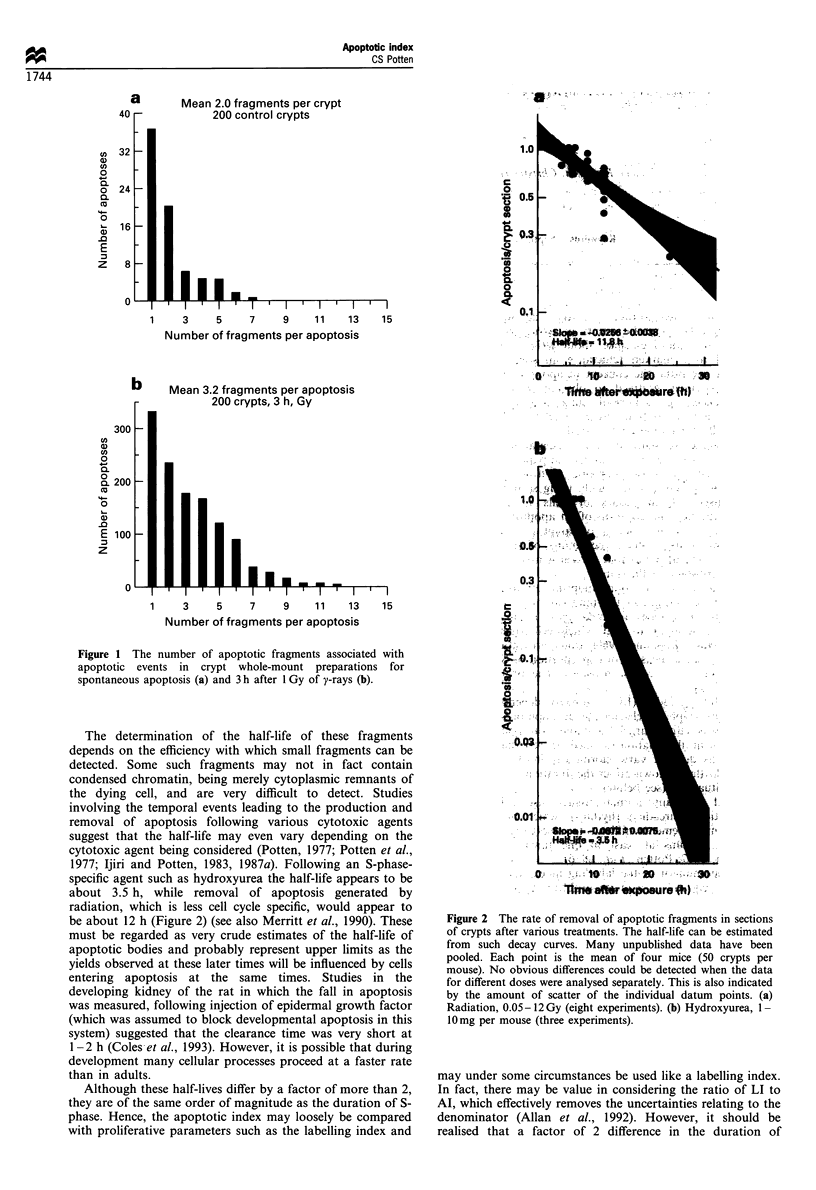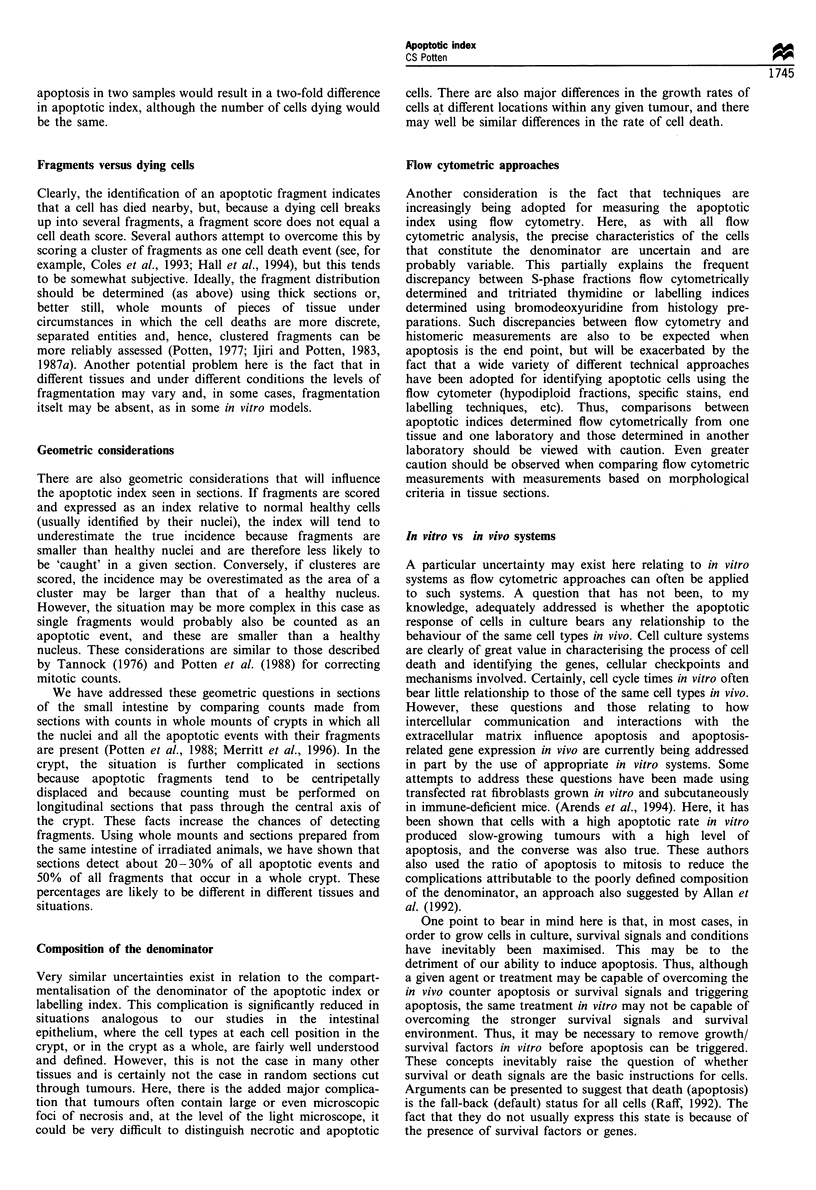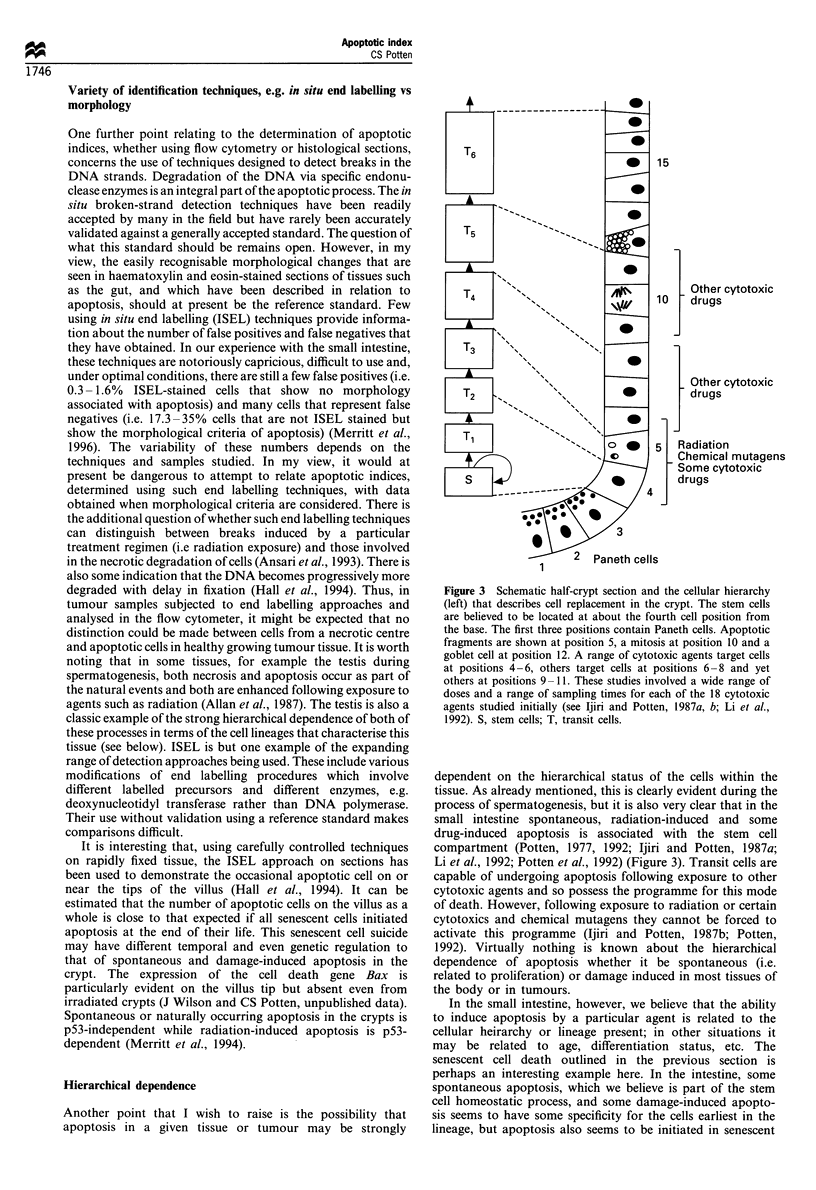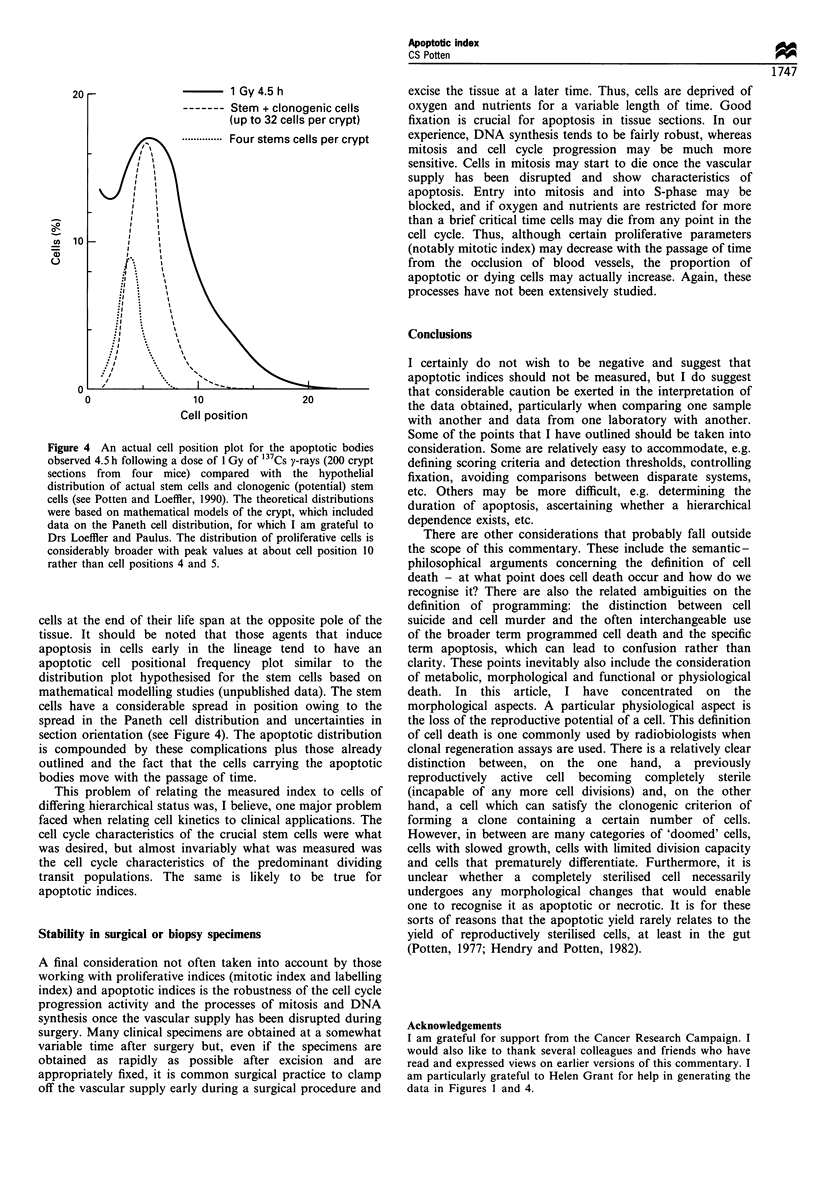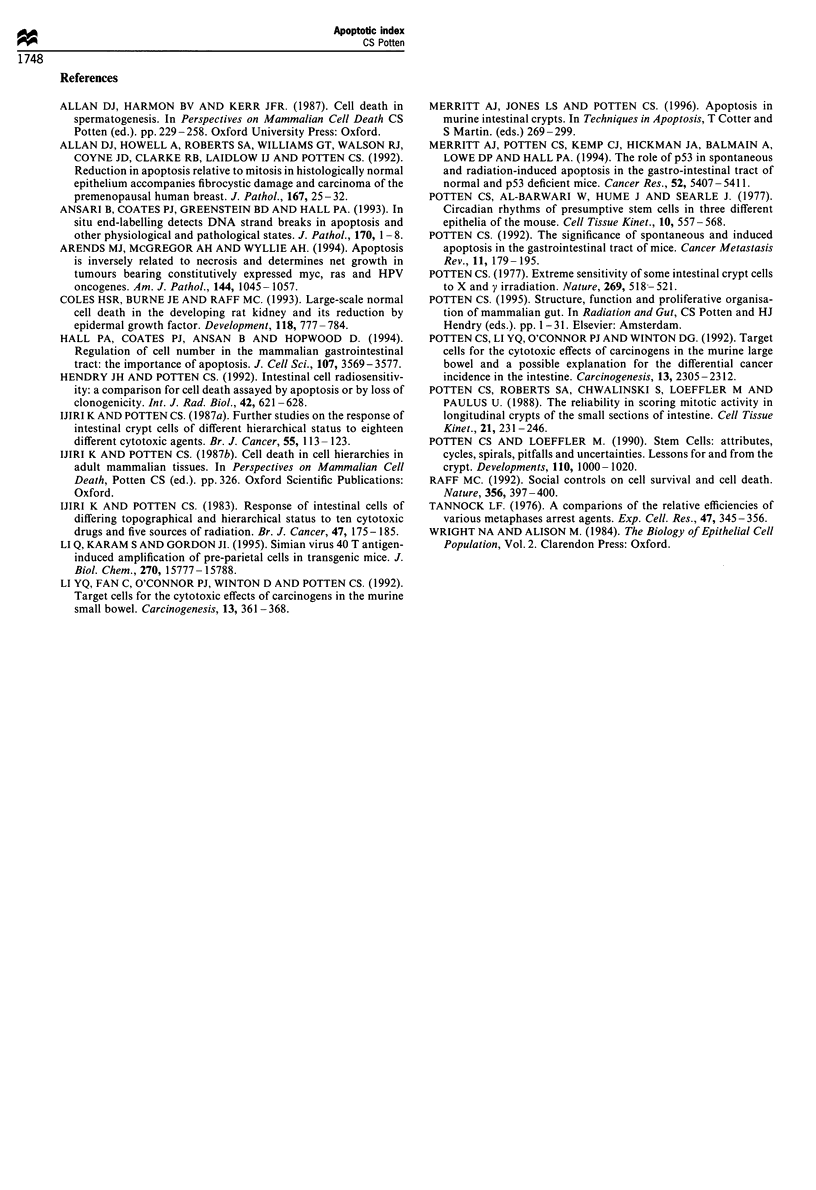# What is an apoptotic index measuring? A commentary.

**DOI:** 10.1038/bjc.1996.624

**Published:** 1996-12

**Authors:** C. S. Potten

**Affiliations:** Paterson Institute for Cancer Research, Christie Hospital NHS Trust, Manchester, UK.


British Journal of Cancer (1996) 74, 1743-1748
© 1996 Stockton Press All rights reserved 0007-0920/96 $12.00

### What is an apoptotic index measuring? A commentary

CS Potten

*Paterson Institute for Cancer Research, Christie Hospital NHS Trust, Wilmslow Road, Manchester M20 9BX, UK.*

**Keywords:** apoptosis; apoptotic index; deviation of apoptosis; apoptotic fragments; *in situ* end labelling

As one who has worked on the identification and quantification of apoptotic events in tissue sections (most notably of the intestine) since about 1974, I had become accustomed to a marked reluctance, in some cases overt hostility, to accepting the concept of apoptotic death. I am therefore extremely gratified to observe that the idea of apoptotis occurring in normal, tumour and cytotoxically exposed tissue is not only widely accepted but has burgeoned into a rapidly expanding field of study.

I have listened to many scientific presentations and read a large number of poster presentations and papers, all of which present measurements of apoptotic yield in a variety of experimental circumstances, usually using sections of tissue, cell suspensions from tissues or cells in culture, and presenting the data in the form of an apoptotic index (AI) (i.e. a measure of the number of apoptotic events or cell deaths expressed as a ratio or percentage of all cells present or all cells counted). I feel that several words of caution are perhaps appropriate at this time.

Those individuals who count apoptotic events and wish to express relative changes in the numbers of these events by way of an apoptotic index are in danger of finding themselves in a similar situation to that which cell kineticists went through in the 1950s and 1960s when presenting labelling indices that were a measure of a proportion of S-phase cells in tissue sections or cell suspensions. The problems faced were as follows:

1. The labelling index (LI) is directly dependent on the number of S-phase cells and the duration of S-phase. The latter was either largely unknown or assumed to be identical in all situations studied. In retrospect, S-phase durations did not vary enormously in the majority of cases. Little is known about the duration of apoptosis in most situations (see below).
2. The LI in many normal tissues also exhibits daily fluctuations (circadian rhythms), which make isolated single measurements difficult to assess. The AI shows similar rhythms at least in one tissue, the intestine (Potten, 1977; Potten *et al.*, 1976).
3. The denominator in the labelling index calculation was, and largely still is, a major problem in that it contains not only cells in other phases of the cell cycle (which is what is assumed by most people) but also non-cycling cells ($G_0$ or quiescent populations), cell types unrelated to the tissue of interest (connective tissue, infiltrating lymphocytes, etc), differentiated non-cycling cells (the functional cells of the tissues of interest) and any dead or dying cells that were not clearly recognisable as dead. Very similar problems associated with the denominator can be expected for AI measurement.
4. These early cell kinetics studies rarely took into account the fact that most renewing tissues, and probably most tumours, have a hierarchical organisation with the predominant dividing transit cells possibly having different cell cycle characteristics from the important and relevant stem or clonogenic compartments. In my view, mainly for this reason, the great expectation in cell kinetics of understanding tumour and normal cell biology so as to facilitate therapy was never fully realised. It is, however, true that most workers in the field of proliferation analyses now have a reasonable appreciation of these problems and limitations. Little is known about the hierarchical dependence of apoptosis except in the gastrointestinal tract.

The points outlined above apply equally well to the determination of an apoptotic index, and, in addition, there may be other considerations that can influence this parameter.

What I would like to attempt here is to consider these problems and others in relation to apoptotic index measurements. Rather than attempt to cover all tissues and cell culture systems I should like to consider just one case, the small intestine, which has been fairly extensively studied over the years. For extensive reviews of the cellular and kinetic organisation see Wright and Alison (1984) and Potten (1995). This could be used as a model for other situations. Inevitably, therefore, I will draw heavily on our own experiences in studying this tissue. I do not wish to create a negative atmosphere concerning apoptotic index but merely to draw attention to potential problems and pitfalls.

### Duration of apoptosis and detection efficiency of small fragments

The duration of the apoptotic process is largely unknown. The microscopically visible part of the process will depend upon the characteristics of the tissue or cell system being studied, the degree of fragmentation that occurs during the apoptotic process, the mechanisms of removal of the apoptotic fragments and the sensitivity of the detection procedures. Some of these aspects have been studied in the gastrointestinal tract, where we have demonstrated that irradiation-induced apoptosis results in the production of, on average, three apoptotic fragments; but there is a wide range of values (Figure 1). These fragments are removed in the intestinal epithelium by phagocytic digestion within healthy neighbouring epithelial cells that are themselves migrating from the crypt to the villus in the small intestine. In other situations the removal is achieved by macrophages. In either case the fragments become smaller with the passage of time and in the intestine also move, or are displaced, along the crypt-villus axis (Ijiri and Potten, 1983, 1987*a*, *b*; Li *et al.*, 1992). However, some fragments may be incorporated into the cytoplasm of stem cells or Paneth cells at the base of the crypt, which either do not move or have a very slow turnover. A few fragments may also be extruded directly into the lumen of the crypt. Hence, the loss or removal process is complex. There are even suggestions from our own unpublished data that the number of fragments generated may vary with the severity or nature of cytotoxic exposure.

Received 12 January 1996; revised 19 June 1996; accepted 27 June 1996

**Figure 1** The number of apoptotic fragments associated with apoptotic events in crypt whole-mount preparations for spontaneous apoptosis (**a**) and 3 h after 1 Gy of $\gamma$-rays (**b**).

The determination of the half-life of these fragments depends on the efficiency with which small fragments can be detected. Some such fragments may not in fact contain condensed chromatin, being merely cytoplasmic remnants of the dying cell, and are very difficult to detect. Studies involving the temporal events leading to the production and removal of apoptosis following various cytotoxic agents suggest that the half-life may even vary depending on the cytotoxic agent being considered (Potten, 1977; Potten *et al.*, 1977; Ijiri and Potten, 1983, 1987*a*). Following an S-phase-specific agent such as hydroxyurea the half-life appears to be about 3.5 h, while removal of apoptosis generated by radiation, which is less cell cycle specific, would appear to be about 12 h (Figure 2) (see also Merritt *et al.*, 1990). These must be regarded as very crude estimates of the half-life of apoptotic bodies and probably represent upper limits as the yields observed at these later times will be influenced by cells entering apoptosis at the same times. Studies in the developing kidney of the rat in which the fall in apoptosis was measured, following injection of epidermal growth factor (which was assumed to block developmental apoptosis in this system) suggested that the clearance time was very short at 1–2 h (Coles *et al.*, 1993). However, it is possible that during development many cellular processes proceed at a faster rate than in adults.

Although these half-lives differ by a factor of more than 2, they are of the same order of magnitude as the duration of S-phase. Hence, the apoptotic index may loosely be compared with proliferative parameters such as the labelling index and

**Figure 2** The rate of removal of apoptotic fragments in sections of crypts after various treatments. The half-life can be estimated from such decay curves. Many unpublished data have been pooled. Each point is the mean of four mice (50 crypts per mouse). No obvious differences could be detected when the data for different doses were analysed separately. This is also indicated by the amount of scatter of the individual datum points. (**a**) Radiation, 0.05–12 Gy (eight experiments). (**b**) Hydroxyurea, 1–10 mg per mouse (three experiments).

may under some circumstances be used like a labelling index. In fact, there may be value in considering the ratio of LI to AI, which effectively removes the uncertainties relating to the denominator (Allan *et al.*, 1992). However, it should be realised that a factor of 2 difference in the duration of apoptosis in two samples would result in a two-fold difference in apoptotic index, although the number of cells dying would be the same.

**Fragments versus dying cells**

Clearly, the identification of an apoptotic fragment indicates that a cell has died nearby, but, because a dying cell breaks up into several fragments, a fragment score does not equal a cell death score. Several authors attempt to overcome this by scoring a cluster of fragments as one cell death event (see, for example, Coles *et al.*, 1993; Hall *et al.*, 1994), but this tends to be somewhat subjective. Ideally, the fragment distribution should be determined (as above) using thick sections or, better still, whole mounts of pieces of tissue under circumstances in which the cell deaths are more discrete, separated entities and, hence, clustered fragments can be more reliably assessed (Potten, 1977; Ijiri and Potten, 1983, 1987*a*). Another potential problem here is the fact that in different tissues and under different conditions the levels of fragmentation may vary and, in some cases, fragmentation itselt may be absent, as in some *in vitro* models.

**Geometric considerations**

There are also geometric considerations that will influence the apoptotic index seen in sections. If fragments are scored and expressed as an index relative to normal healthy cells (usually identified by their nuclei), the index will tend to underestimate the true incidence because fragments are smaller than healthy nuclei and are therefore less likely to be 'caught' in a given section. Conversely, if clusteres are scored, the incidence may be overestimated as the area of a cluster may be larger than that of a healthy nucleus. However, the situation may be more complex in this case as single fragments would probably also be counted as an apoptotic event, and these are smaller than a healthy nucleus. These considerations are similar to those described by Tannock (1976) and Potten *et al.* (1988) for correcting mitotic counts.

We have addressed these geometric questions in sections of the small intestine by comparing counts made from sections with counts in whole mounts of crypts in which all the nuclei and all the apoptotic events with their fragments are present (Potten *et al.*, 1988; Merritt *et al.*, 1996). In the crypt, the situation is further complicated in sections because apoptotic fragments tend to be centripetally displaced and because counting must be performed on longitudinal sections that pass through the central axis of the crypt. These facts increase the chances of detecting fragments. Using whole mounts and sections prepared from the same intestine of irradiated animals, we have shown that sections detect about 20–30% of all apoptotic events and 50% of all fragments that occur in a whole crypt. These percentages are likely to be different in different tissues and situations.

**Composition of the denominator**

Very similar uncertainties exist in relation to the compartmentalisation of the denominator of the apoptotic index or labelling index. This complication is significantly reduced in situations analogous to our studies in the intestinal epithelium, where the cell types at each cell position in the crypt, or in the crypt as a whole, are fairly well understood and defined. However, this is not the case in many other tissues and is certainly not the case in random sections cut through tumours. Here, there is the added major complication that tumours often contain large or even microscopic foci of necrosis and, at the level of the light microscope, it could be very difficult to distinguish necrotic and apoptotic cells. There are also major differences in the growth rates of cells at different locations within any given tumour, and there may well be similar differences in the rate of cell death.

**Flow cytometric approaches**

Another consideration is the fact that techniques are increasingly being adopted for measuring the apoptotic index using flow cytometry. Here, as with all flow cytometric analysis, the precise characteristics of the cells that constitute the denominator are uncertain and are probably variable. This partially explains the frequent discrepancy between S-phase fractions flow cytometrically determined and tritiated thymidine or labelling indices determined using bromodeoxyuridine from histology preparations. Such discrepancies between flow cytometry and histomeric measurements are also to be expected when apoptosis is the end point, but will be exacerbated by the fact that a wide variety of different technical approaches have been adopted for identifying apoptotic cells using the flow cytometer (hypodiploid fractions, specific stains, end labelling techniques, etc). Thus, comparisons between apoptotic indices determined flow cytometrically from one tissue and one laboratory and those determined in another laboratory should be viewed with caution. Even greater caution should be observed when comparing flow cytometric measurements with measurements based on morphological criteria in tissue sections.

***In vitro* vs *in vivo* systems**

A particular uncertainty may exist here relating to *in vitro* systems as flow cytometric approaches can often be applied to such systems. A question that has not been, to my knowledge, adequately addressed is whether the apoptotic response of cells in culture bears any relationship to the behaviour of the same cell types *in vivo*. Cell culture systems are clearly of great value in characterising the process of cell death and identifying the genes, cellular checkpoints and mechanisms involved. Certainly, cell cycle times *in vitro* often bear little relationship to those of the same cell types *in vivo*. However, these questions and those relating to how intercellular communication and interactions with the extracellular matrix influence apoptosis and apoptosis-related gene expression *in vivo* are currently being addressed in part by the use of appropriate *in vitro* systems. Some attempts to address these questions have been made using transfected rat fibroblasts grown *in vitro* and subcutaneously in immune-deficient mice. (Arends *et al.*, 1994). Here, it has been shown that cells with a high apoptotic rate *in vitro* produced slow-growing tumours with a high level of apoptosis, and the converse was also true. These authors also used the ratio of apoptosis to mitosis to reduce the complications attributable to the poorly defined composition of the denominator, an approach also suggested by Allan *et al.* (1992).

One point to bear in mind here is that, in most cases, in order to grow cells in culture, survival signals and conditions have inevitably been maximised. This may be to the detriment of our ability to induce apoptosis. Thus, although a given agent or treatment may be capable of overcoming the *in vivo* counter apoptosis or survival signals and triggering apoptosis, the same treatment *in vitro* may not be capable of overcoming the stronger survival signals and survival environment. Thus, it may be necessary to remove growth/survival factors *in vitro* before apoptosis can be triggered. These concepts inevitably raise the question of whether survival or death signals are the basic instructions for cells. Arguments can be presented to suggest that death (apoptosis) is the fall-back (default) status for all cells (Raff, 1992). The fact that they do not usually express this state is because of the presence of survival factors or genes.

**Variety of identification techniques, e.g. *in situ* end labelling vs morphology**

One further point relating to the determination of apoptotic indices, whether using flow cytometry or histological sections, concerns the use of techniques designed to detect breaks in the DNA strands. Degradation of the DNA via specific endonuclease enzymes is an integral part of the apoptotic process. The *in situ* broken-strand detection techniques have been readily accepted by many in the field but have rarely been accurately validated against a generally accepted standard. The question of what this standard should be remains open. However, in my view, the easily recognisable morphological changes that are seen in haematoxylin and eosin-stained sections of tissues such as the gut, and which have been described in relation to apoptosis, should at present be the reference standard. Few using *in situ* end labelling (ISEL) techniques provide information about the number of false positives and false negatives that they have obtained. In our experience with the small intestine, these techniques are notoriously capricious, difficult to use and, under optimal conditions, there are still a few false positives (i.e. 0.3–1.6% ISEL-stained cells that show no morphology associated with apoptosis) and many cells that represent false negatives (i.e. 17.3–35% cells that are not ISEL stained but show the morphological criteria of apoptosis) (Merritt *et al.*, 1996). The variability of these numbers depends on the techniques and samples studied. In my view, it would at present be dangerous to attempt to relate apoptotic indices, determined using such end labelling techniques, with data obtained when morphological criteria are considered. There is the additional question of whether such end labelling techniques can distinguish between breaks induced by a particular treatment regimen (i.e radiation exposure) and those involved in the necrotic degradation of cells (Ansari *et al.*, 1993). There is also some indication that the DNA becomes progressively more degraded with delay in fixation (Hall *et al.*, 1994). Thus, in tumour samples subjected to end labelling approaches and analysed in the flow cytometer, it might be expected that no distinction could be made between cells from a necrotic centre and apoptotic cells in healthy growing tumour tissue. It is worth noting that in some tissues, for example the testis during spermatogenesis, both necrosis and apoptosis occur as part of the natural events and both are enhanced following exposure to agents such as radiation (Allan *et al.*, 1987). The testis is also a classic example of the strong hierarchical dependence of both of these processes in terms of the cell lineages that characterise this tissue (see below). ISEL is but one example of the expanding range of detection approaches being used. These include various modifications of end labelling procedures which involve different labelled precursors and different enzymes, e.g. deoxynucleotidyl transferase rather than DNA polymerase. Their use without validation using a reference standard makes comparisons difficult.

It is interesting that, using carefully controlled techniques on rapidly fixed tissue, the ISEL approach on sections has been used to demonstrate the occasional apoptotic cell on or near the tips of the villus (Hall *et al.*, 1994). It can be estimated that the number of apoptotic cells on the villus as a whole is close to that expected if all senescent cells initiated apoptosis at the end of their life. This senescent cell suicide may have different temporal and even genetic regulation to that of spontaneous and damage-induced apoptosis in the crypt. The expression of the cell death gene *Bax* is particularly evident on the villus tip but absent even from irradiated crypts (J Wilson and CS Potten, unpublished data). Spontaneous or naturally occurring apoptosis in the crypts is p53-independent while radiation-induced apoptosis is p53-dependent (Merritt *et al.*, 1994).

**Hierarchical dependence**

Another point that I wish to raise is the possibility that apoptosis in a given tissue or tumour may be strongly dependent on the hierarchical status of the cells within the tissue. As already mentioned, this is clearly evident during the process of spermatogenesis, but it is also very clear that in the small intestine spontaneous, radiation-induced and some drug-induced apoptosis is associated with the stem cell compartment (Potten, 1977, 1992; Ijiri and Potten, 1987*a*; Li *et al.*, 1992; Potten *et al.*, 1992) (Figure 3). Transit cells are capable of undergoing apoptosis following exposure to other cytotoxic agents and so possess the programme for this mode of death. However, following exposure to radiation or certain cytotoxics and chemical mutagens they cannot be forced to activate this programme (Ijiri and Potten, 1987b; Potten, 1992). Virtually nothing is known about the hierarchical dependence of apoptosis whether it be spontaneous (i.e. related to proliferation) or damage induced in most tissues of the body or in tumours.

In the small intestine, however, we believe that the ability to induce apoptosis by a particular agent is related to the cellular heirarchy or lineage present; in other situations it may be related to age, differentiation status, etc. The senescent cell death outlined in the previous section is perhaps an interesting example here. In the intestine, some spontaneous apoptosis, which we believe is part of the stem cell homeostatic process, and some damage-induced apoptosis seems to have some specificity for the cells earliest in the lineage, but apoptosis also seems to be initiated in senescent

**Figure 3** Schematic half-crypt section and the cellular hierarchy (left) that describes cell replacement in the crypt. The stem cells are believed to be located at about the fourth cell position from the base. The first three positions contain Paneth cells. Apoptotic fragments are shown at position 5, a mitosis at position 10 and a goblet cell at position 12. A range of cytotoxic agents target cells at positions 4–6, others target cells at positions 6–8 and yet others at positions 9–11. These studies involved a wide range of doses and a range of sampling times for each of the 18 cytotoxic agents studied initially (see Ijiri and Potten, 1987*a*, *b*; Li *et al.*, 1992). S, stem cells; T, transit cells.

**Figure 4** An actual cell position plot for the apoptotic bodies observed 4.5 h following a dose of 1 Gy of $^{137}Cs$ $\gamma$-rays (200 crypt sections from four mice) compared with the hypothelial distribution of actual stem cells and clonogenic (potential) stem cells (see Potten and Loeffler, 1990). The theoretical distributions were based on mathematical models of the crypt, which included data on the Paneth cell distribution, for which I am grateful to Drs Loeffler and Paulus. The distribution of proliferative cells is considerably broader with peak values at about cell position 10 rather than cell positions 4 and 5.

cells at the end of their life span at the opposite pole of the tissue. It should be noted that those agents that induce apoptosis in cells early in the lineage tend to have an apoptotic cell positional frequency plot similar to the distribution plot hypothesised for the stem cells based on mathematical modelling studies (unpublished data). The stem cells have a considerable spread in position owing to the spread in the Paneth cell distribution and uncertainties in section orientation (see Figure 4). The apoptotic distribution is compounded by these complications plus those already outlined and the fact that the cells carrying the apoptotic bodies move with the passage of time.

This problem of relating the measured index to cells of differing hierarchical status was, I believe, one major problem faced when relating cell kinetics to clinical applications. The cell cycle characteristics of the crucial stem cells were what was desired, but almost invariably what was measured was the cell cycle characteristics of the predominant dividing transit populations. The same is likely to be true for apoptotic indices.

### Stability in surgical or biopsy specimens

A final consideration not often taken into account by those working with proliferative indices (mitotic index and labelling index) and apoptotic indices is the robustness of the cell cycle progression activity and the processes of mitosis and DNA synthesis once the vascular supply has been disrupted during surgery. Many clinical specimens are obtained at a somewhat variable time after surgery but, even if the specimens are obtained as rapidly as possible after excision and are appropriately fixed, it is common surgical practice to clamp off the vascular supply early during a surgical procedure and excise the tissue at a later time. Thus, cells are deprived of oxygen and nutrients for a variable length of time. Good fixation is crucial for apoptosis in tissue sections. In our experience, DNA synthesis tends to be fairly robust, whereas mitosis and cell cycle progression may be much more sensitive. Cells in mitosis may start to die once the vascular supply has been disrupted and show characteristics of apoptosis. Entry into mitosis and into S-phase may be blocked, and if oxygen and nutrients are restricted for more than a brief critical time cells may die from any point in the cell cycle. Thus, although certain proliferative parameters (notably mitotic index) may decrease with the passage of time from the occlusion of blood vessels, the proportion of apoptotic or dying cells may actually increase. Again, these processes have not been extensively studied.

### Conclusions

I certainly do not wish to be negative and suggest that apoptotic indices should not be measured, but I do suggest that considerable caution be exerted in the interpretation of the data obtained, particularly when comparing one sample with another and data from one laboratory with another. Some of the points that I have outlined should be taken into consideration. Some are relatively easy to accommodate, e.g. defining scoring criteria and detection thresholds, controlling fixation, avoiding comparisons between disparate systems, etc. Others may be more difficult, e.g. determining the duration of apoptosis, ascertaining whether a hierarchical dependence exists, etc.

There are other considerations that probably fall outside the scope of this commentary. These include the semantic–philosophical arguments concerning the definition of cell death – at what point does cell death occur and how do we recognise it? There are also the related ambiguities on the definition of programming: the distinction between cell suicide and cell murder and the often interchangeable use of the broader term programmed cell death and the specific term apoptosis, which can lead to confusion rather than clarity. These points inevitably also include the consideration of metabolic, morphological and functional or physiological death. In this article, I have concentrated on the morphological aspects. A particular physiological aspect is the loss of the reproductive potential of a cell. This definition of cell death is one commonly used by radiobiologists when clonal regeneration assays are used. There is a relatively clear distinction between, on the one hand, a previously reproductively active cell becoming completely sterile (incapable of any more cell divisions) and, on the other hand, a cell which can satisfy the clonogenic criterion of forming a clone containing a certain number of cells. However, in between are many categories of 'doomed' cells, cells with slowed growth, cells with limited division capacity and cells that prematurely differentiate. Furthermore, it is unclear whether a completely sterilised cell necessarily undergoes any morphological changes that would enable one to recognise it as apoptotic or necrotic. It is for these sorts of reasons that the apoptotic yield rarely relates to the yield of reproductively sterilised cells, at least in the gut (Potten, 1977; Hendry and Potten, 1982).

**Acknowledgements**
I am grateful for support from the Cancer Research Campaign. I would also like to thank several colleagues and friends who have read and expressed views on earlier versions of this commentary. I am particularly grateful to Helen Grant for help in generating the data in Figures 1 and 4.

### References

ALLAN DJ, HARMON BV AND KERR JFR. (1987). Cell death in spermatogenesis. In *Perspectives on Mammalian Cell Death* CS Potten (ed.). pp. 229–258. Oxford University Press: Oxford.

ALLAN DJ, HOWELL A, ROBERTS SA, WILLIAMS GT, WALSON RJ, COYNE JD, CLARKE RB, LAIDLOW IJ AND POTTEN CS. (1992). Reduction in apoptosis relative to mitosis in histologically normal epithelium accompanies fibrocystic damage and carcinoma of the premenopausal human breast. *J. Pathol.*, **167,** 25–32.

ANSARI B, COATES PJ, GREENSTEIN BD AND HALL PA. (1993). In situ end-labelling detects DNA strand breaks in apoptosis and other physiological and pathological states. *J. Pathol.*, **170,** 1–8.

ARENDS MJ, MCGREGOR AH AND WYLLIE AH. (1994). Apoptosis is inversely related to necrosis and determines net growth in tumours bearing constitutively expressed myc, ras and HPV oncogenes. *Am. J. Pathol.*, **144,** 1045–1057.

COLES HSR, BURNE JE AND RAFF MC. (1993). Large-scale normal cell death in the developing rat kidney and its reduction by epidermal growth factor. *Development*, **118,** 777–784.

HALL PA, COATES PJ, ANSAN B AND HOPWOOD D. (1994). Regulation of cell number in the mammalian gastrointestinal tract: the importance of apoptosis. *J. Cell Sci.*, **107,** 3569–3577.

HENDRY JH AND POTTEN CS. (1992). Intestinal cell radiosensitivity: a comparison for cell death assayed by apoptosis or by loss of clonogenicity. *Int. J. Rad. Biol.*, **42,** 621–628.

IJIRI K AND POTTEN CS. (1987*a*). Further studies on the response of intestinal crypt cells of different hierarchical status to eighteen different cytotoxic agents. *Br. J. Cancer*, **55,** 113–123.

IJIRI K AND POTTEN CS. (1987*b*). Cell death in cell hierarchies in adult mammalian tissues. In *Perspectives on Mammalian Cell Death*, Potten CS (ed.). pp. 326. Oxford Scientific Publications: Oxford.

IJIRI K AND POTTEN CS. (1983). Response of intestinal cells of differing topographical and hierarchical status to ten cytotoxic drugs and five sources of radiation. *Br. J. Cancer*, **47,** 175–185.

LI Q, KARAM S AND GORDON JI. (1995). Simian virus 40 T antigen-induced amplification of pre-parietal cells in transgenic mice. *J. Biol. Chem.*, **270,** 15777–15788.

LI YQ, FAN C, O'CONNOR PJ, WINTON D AND POTTEN CS. (1992). Target cells for the cytotoxic effects of carcinogens in the murine small bowel. *Carcinogenesis*, **13,** 361–368.

MERRITT AJ, JONES LS AND POTTEN CS. (1996). Apoptosis in murine intestinal crypts. In *Techniques in Apoptosis*, T Cotter and S Martin. (eds.) 269–299.

MERRITT AJ, POTTEN CS, KEMP CJ, HICKMAN JA, BALMAIN A, LOWE DP AND HALL PA. (1994). The role of p53 in spontaneous and radiation-induced apoptosis in the gastro-intestinal tract of normal and p53 deficient mice. *Cancer Res.*, **52,** 5407–5411.

POTTEN CS, AL-BARWARI W, HUME J AND SEARLE J. (1977). Circadian rhythms of presumptive stem cells in three different epithelia of the mouse. *Cell Tissue Kinet.*, **10,** 557–568.

POTTEN CS. (1992). The significance of spontaneous and induced apoptosis in the gastrointestinal tract of mice. *Cancer Metastasis Rev.*, **11,** 179–195.

POTTEN CS. (1977). Extreme sensitivity of some intestinal crypt cells to X and $\gamma$ irradiation. *Nature*, **269,** 518–521.

POTTEN CS. (1995). Structure, function and proliferative organisation of mammalian gut. In *Radiation and Gut*, CS Potten and HJ Hendry (eds.). pp. 1–31. Elsevier: Amsterdam.

POTTEN CS, LI YQ, O'CONNOR PJ AND WINTON DG. (1992). Target cells for the cytotoxic effects of carcinogens in the murine large bowel and a possible explanation for the differential cancer incidence in the intestine. *Carcinogenesis*, **13,** 2305–2312.

POTTEN CS, ROBERTS SA, CHWALINSKI S, LOEFFLER M AND PAULUS U. (1988). The reliability in scoring mitotic activity in longitudinal crypts of the small sections of intestine. *Cell Tissue Kinet.*, **21,** 231–246.

POTTEN CS AND LOEFFLER M. (1990). Stem Cells: attributes, cycles, spirals, pitfalls and uncertainties. Lessons for and from the crypt. *Developments*, **110,** 1000–1020.

RAFF MC. (1992). Social controls on cell survival and cell death. *Nature*, **356,** 397–400.

TANNOCK LF. (1976). A comparions of the relative efficiencies of various metaphases arrest agents. *Exp. Cell. Res.*, **47,** 345–356.

WRIGHT NA AND ALISON M. (1984). *The Biology of Epithelial Cell Population*, Vol. 2. Clarendon Press: Oxford.